# Supplementary material for: The validity and reliability of the Test of Memory Strategies among Italian healthy adults
Source: PeerJ. 2022 Sep 29;10:e14059. doi: 10.7717/peerj.14059 (PMC9527021; doi:10.7717/peerj.14059)
Supplement: Supplemental Information 1 [file peerj-10-14059-s001.docx]

**Supplementary Material**

Table 1 Original version of the Test of the Memory Strategies

| Aprendizaje incidental | No relación Semántica | Semántica-no consciente  2 categorìas - desordenadas | Semántica-no consciente  2 categorìas - ordenadas | Semántica-consciente  2 categorìas - ordenadas |
| --- | --- | --- | --- | --- |
| 1. Bloqueo 2. Pueblo 3. Química 4. Polémica 5. Falta 6. Capricho 7. Momento 8. Público 9. Culpa 10. Estrella   1.  2.  3.  4.  5.  6.  7.  8.  9.  10. | 1. Esencia 2. Sentido 3. Consejo 4. Grado 5. Forma 6. Ejemplo 7. Impureza 8. Fe 9. Tonelada 10. Precio   1.  2.  3.  4.  5.  6.  7.  8.  9.  10. | 1. **Roble** 2. Sillón 3. Cama 4. **Nogal** 5. Perchero 6. **Melocotonero** 7. **Sauce** 8. Mesa 9. **Pino** 10. Armario   1.  2.  3.  4.  5.  6.  7.  8.  9.  10. | 1. **Automóvil** 2. **Tren** 3. **Autobús** 4. **Helicóptero** 5. **Tranvía** 6. Martillo 7. Tenazas 8. Serrucho 9. Destornillador 10. Alicate   1.  2.  3.  4.  5.  6.  7.  8.  9.  10. | 1. **Atletismo** 2. **Futbol** 3. **Natación** 4. **Rugby** 5. **Balonmano** 6. Repollo 7. Apio 8. Judías 9. Alcachofas 10. Berenjena   1.  2.  3.  4.  5.  6.  7.  8.  9.  10. |
| Total:  Intrusiones: | Total:  Intrusiones: | Total:  Total Categoría 1:  Total Categoría 2:  Intrusiones: | Total:  Total Categoría 1:  Total Categoría 2:  Intrusiones: | Total:  Total Categoría 1:  Total Categoría 2:  Intrusiones: |

| Apprendimento incidentale | No relazione semantica | Semantica-non cosciente  2 categorie - disordinate | Semantica-non cosciente  2 categorie - ordinate | Semantica-cosciente  2 categorie - ordinate |
| --- | --- | --- | --- | --- |
| 1. Blocco 2. Paese 3. Chimica 4. Polemica 5. Errore 6. Capriccio 7. Momento 8. Pubblico 9. Colpa 10. Stella   1.  2.  3.  4.  5.  6.  7.  8.  9.  10. | 1. Essenza 2. Senso 3. Consiglio 4. Grado 5. Forma 6. Esempio 7. Impurità 8. Fede 9. Tonnellata 10. Prezzo   1.  2.  3.  4.  5.  6.  7.  8.  9.  10. | 1. **Quercia** 2. Poltrona 3. Letto 4. **Noce** 5. Attaccapanni 6. **Pesco** 7. **Salice** 8. Tavolo 9. **Pino** 10. Armadio   1.  2.  3.  4.  5.  6.  7.  8.  9.  10. | 1. **Automobile** 2. **Treno** 3. **Autobus** 4. **Elicottero** 5. **Tram** 6. Martello 7. Tenaglie 8. Seghetto 9. Cacciavite 10. Pinza   1.  2.  3.  4.  5.  6.  7.  8.  9.  10. | 1. **Atletica** 2. **Calcio** 3. **Nuoto** 4. **Rugby** 5. **Pallamano** 6. Cavolo 7. Sedano 8. Fagioli 9. Carciofi 10. Melanzana   1.  2.  3.  4.  5.  6.  7.  8.  9.  10. |
| Totale:  Intrusioni: | Totale:  Intrusioni: | Totale:  Totale Categoria 1:  Totale Categoria 2:  Intrusioni: | Total:  Total Categoria 1:  Total Categoria 2:  Intrusioni: | Total:  Total Categoria 1:  Total Categoria 2:  Intrusioni: |

Table 2 Translate version of the Test of the Memory Strategies
