# Supplementary figures and images for: The validity and reliability of the Test of Memory Strategies among Italian healthy adults
Source: PeerJ. 2022 Sep 29;10:e14059. doi: 10.7717/peerj.14059 (PMC9527021; doi:10.7717/peerj.14059)

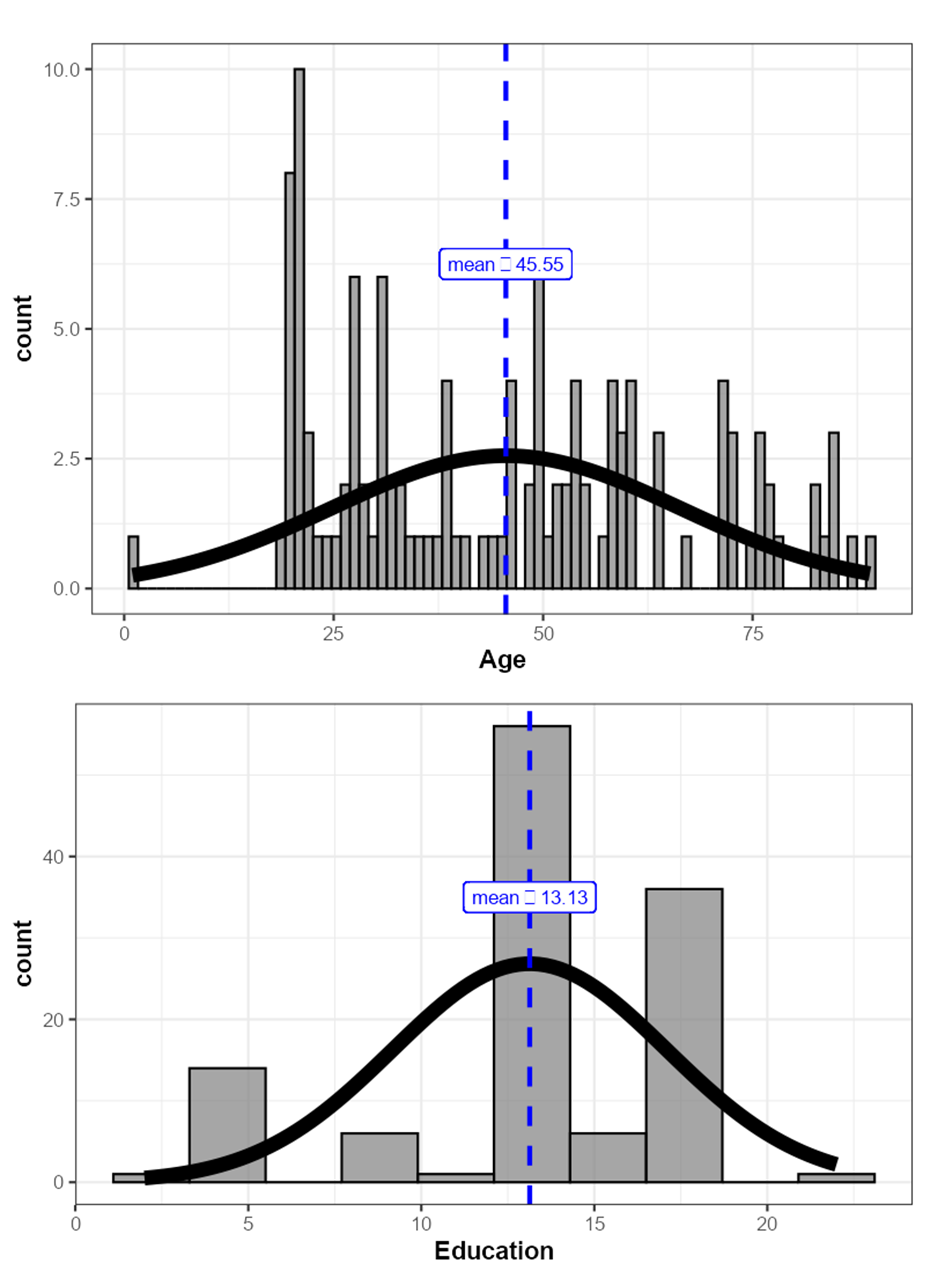

Supplement: Supplemental Information 2 [file peerj-10-14059-s002.png]
